# Analysis of regulator of G-protein signalling 2 (RGS2) expression and function during prostate cancer progression

**DOI:** 10.1038/s41598-018-35332-4

**Published:** 2018-11-22

**Authors:** Anna Linder, Malin Hagberg Thulin, Jan-Erik Damber, Karin Welén

**Affiliations:** 1 Department of Urology, Sahlgrenska Cancer Center, Institute of Clinical Sciences, Sahlgrenska Academy at the University of Gothenburg, Göteborg, Sweden; 2 Department of Radiation Sciences, Department of Oncology, Umeå University, Umeå, Sweden

## Abstract

Prostate cancer (PC) represents the second highest cancer-related mortality among men and the call for biomarkers for early discrimination between aggressive and indolent forms is essential. Downregulation of Regulator of G-protein signaling 2 (RGS2) has been shown in PC, however the underlying mechanism has not been described. Aberrant RGS2 expression has also been reported for other carcinomas in association to both positive and negative prognosis. In this study, we assessed RGS2 expression during PC progression in terms of regulation and impact on tumour phenotype and evaluated its prognostic value. Our experimental data suggest that the RGS2 downregulation seen in early PC is caused by hypoxia. In line with the common indolent phenotype of a primary PC, knockdown of RGS2 induced epithelial features and impaired metastatic properties. However, increased STAT3, TWIST1 and decreased E-cadherin expression suggest priming for EMT. Additionally, improved tumour cell survival and increased BCL-2 expression linked decreased RGS2 levels to fundamental tumour advantages. In contrast, high RGS2 levels in advanced PC were correlated to poor patient survival and a positive metastatic status. This study describes novel roles for RGS2 during PC progression and suggests a prognostic potential discriminating between indolent and metastatic forms of PC.

## Introduction

Prostate cancer (PC) is the second most common diagnosed cancer in men worldwide and associated with the second highest cancer-related mortality^1^. Primary PC is often non-invasive, with an indolent clinical course, however due to the heterogeneous nature of PC several studies reveal unpredictability regarding progression of localized untreated PC also among low-risk tumours^2–6^. Today, diagnosis and stratification of patients into treatment groups are based on levels of prostate specific antigen (PSA), Gleason score (GS) and T-stage. These variables are widely successful; however enhanced understanding of cancer biology for further clinical improvement in terms of diagnosis and treatment prediction is necessary.

In this study, we assessed the regulator of G-protein signalling 2 (RGS2) in association to PC progression. RGS2 is a protein with various modes of action; it was first described as a G0/G1 switch regulatory gene^7^ and has been shown to negatively regulate protein translation^8,9^ and assist gene transcription in association with the transcription factor hypoxia-inducible factor 1 (HIF1)^10^. Despite several described functions, RGS2 has mostly been considered for its inhibitory effect on G-protein signalling via its GTPase-activating protein (GAP) activity^11–13^. The diversity of the RGS2 protein can be explained by the presence of distinct functional isoforms^14^. Taken together, RGS2 has the ability to affect various basic cellular processes.

Wolff *et al*. described downregulation of RGS2 in PC compared to normal or benign tissue and suggested that decreased levels of RGS2 is associated with tumour progression^15^. Aberrant RGS2 expression has also been described for other types of carcinomas in association to both positive and negative prognosis^16–21^. These contradictory data reflect the diverse action of RGS2 and confirm context dependent expression and function.

Aberrant RGS2 expression has been suggested in response to hypoxia in several cell- and tissue types^22,23^. Hypoxia impacts numerous biological processes linked to tumour progression and survival^24,25^. In the increasingly hypoxic tumour environment, cells with the potential to evade cell death are enriched by the induction of anti-apoptotic proteins such as B-cell lymphoma 2 (BCL-2)^26–28^ and hypoxia-inducible factor 1-alpha (HIF1A)^29^. Both HIF1A and BCL-2 has been associated with epithelial-mesenchymal transition (EMT)^30–33^. EMT defines the progression from a polarized epithelial phenotype to a mesenchymal cell phenotype. It is a dynamic and reversible biological process with intermediate states^34–39^. Central signalling pathways like the signal transducer and activator of transcription 3 (STAT3) and phosphoinositide 3-kinase (PI3K)/AKT pathways has also been linked to EMT and metastasis^40–42^. Increased STAT3 activation has been reported for a vast majority of PC^43,44^ and also induced PI3K/AKT signalling is a common aberration associated with tumour progression and advanced PC^41,45–47^.

Taken together, the suggested associations between RGS2 downregulation and PC development^15^, hypoxia^22,23^ and EMT^48^ implies further studies of RGS2 and its role during tumour progression. The regulation and biological significance of aberrant RGS2 expression in hormone-naïve primary PC have not been fully clarified. Therefore, this study was designed to evaluate RGS2 expression in two separate patient cohorts and to assess the correlation to clinicopathological factors. The regulation of RGS2 was also experimentally assessed together with its impact on tumour cell phenotype and effects on relevant signalling pathways corresponding to the phenotypic outcome of aberrant RGS2 expression in PC.

## Results

### Initial downregulation of RGS2 during PC development

**Figure 1:**
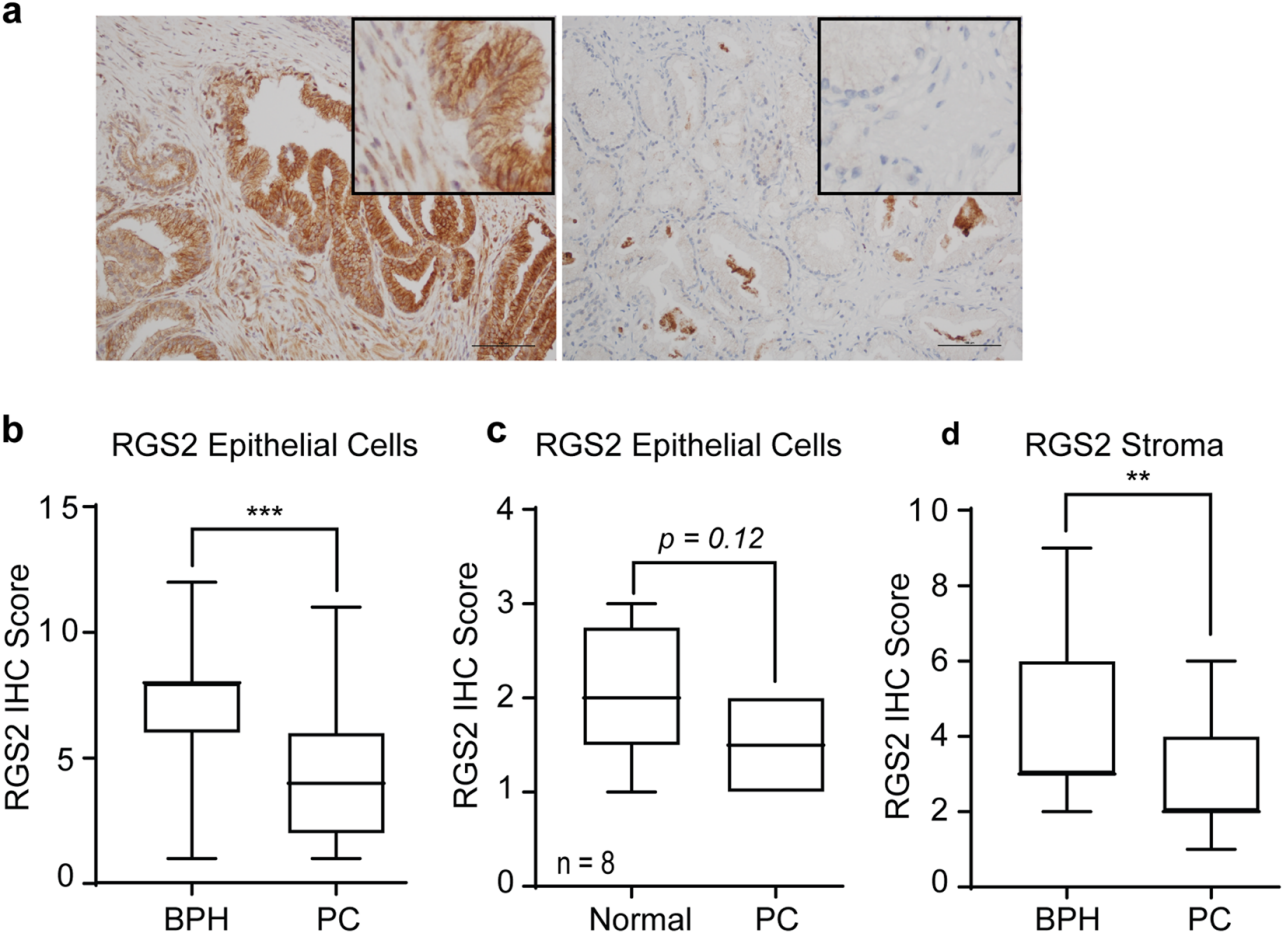
RGS2 protein expression in patient specimens. RGS2 Immunohistochemistry (IHC) staining of TURP specimens. (**a**) Representative images of RGS2 expression in BPH and hormone-naïve PC specimens in stage T1b, showing the expression pattern of epithelial and stromal cells. Bar = 100 µm. (**b**) Quantitative comparisons of RGS2 expression of epithelial cells of BPH (*n* = 36) and PC (*n* = 28) (p < 0.00001, by Mann-Whitney test). (**c**) Intra sectional RGS2 difference in RGS2 expression between PC areas and adjacent normal areas (*n* = 8) (p = 0.12, Wilcoxon W paired test). (**d**) Quantitative comparison of RGS2 staining in BPH (*n* = 36) and PC associated stroma (*n* = 27) (p = 0.007, Mann-Whitney test).

RGS2 expression was immunohistochemically evaluated and compared between low grade PC in stage T1b and benign or normal prostate tissue. The data shows that RGS2 was expressed at high levels in luminal epithelial cells of benign origin but decreased in PC cells (Fig. 1a,b). For BPH samples, 52.8% (19/36) showed high RGS2 protein expression (section score 8 or above) compared to only 17.9% (5/28) of the PC specimens. In comparison 39.3% (11/28) of the PC specimens showed negligible RGS2 expression (IHC score of 2 or less) compared to 5.6% (2/36) in the BPH group. However statistically non-significant, intra-sectional differences of RGS2 expression indicated that malignant areas expressed decreased RGS2 levels compared to adjacent non-cancerous tissue though the median value where low for both groups (Fig. 1c). Further analysis showed that tumour stroma expressed decreased levels of RGS2 compared to BPH associated stroma (Fig. 1d). There was additionally a positive intra-sectional correlation between stromal staining and epithelial staining (Spearman’s rho 0.323, p = 0.001). There was no correlation between any of the clinicopathological factors (listed in Supplementary Table S1) and RGS2 staining (Spearman’s rho).

### RGS2 expression is suppressed by hypoxia

**Figure 2:**
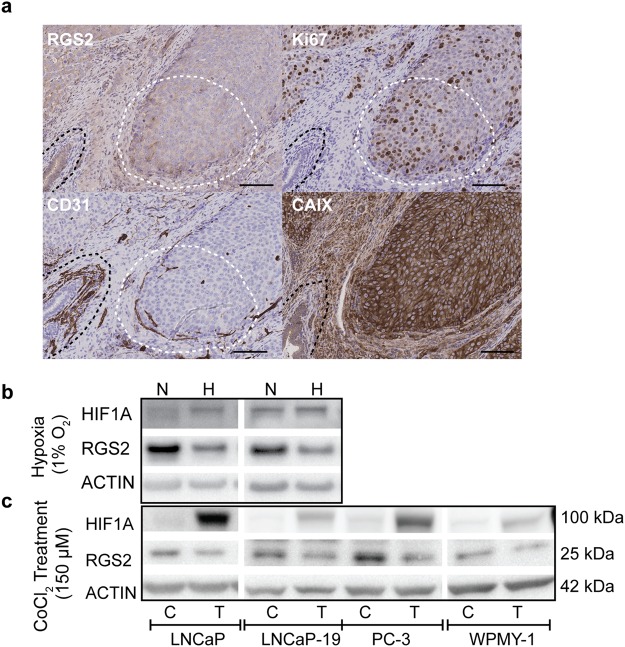
RGS2 expression is inhibited by hypoxia. (**a**) Overview of RGS2 expression in LNCaP descending mouse tumours representative of RGS2 expression in correlation to blood vessels, proliferation and hypoxia (Bar = 100 µm). (**b**) Representative western blot from cell lysates obtained from cells cultured in a hypoxic atmosphere (H) (1% O_2_) for 72 hours and corresponding normoxic controls (N) (21% O_2_). HIF1A controls for hypoxic response and β-actin included for protein loading. (**c**) Representative WB from cell lysates obtained after 72 hours of CoCl_2_ treatment (T) and respective control (C). The blots were cropped. LNCaP and LNCaP-19 was run on the same gel for both experiments (Hypoxia and CoCl_2_) and PC-3 and WPMY were run on the same gel. All gels were run under the same conditions. The original pictures of the full-length western blots can be found in Supplementary Fig. S3.

The description of HIF1A as an early marker for tumour development, lead to the assumption that hypoxia could cause decreased RGS2 expression in early PC. In agreement, RGS2 IHC staining of LNCaP orthotopic xenografts (Fig. 2a) showed high levels of RGS2 in proximity to blood vessels and well vascularised loci (white dashed line) but gradually decreased RGS2 expression towards hypoxic areas. In accordance with data from the patient material, the normal prostatic epithelium expressed high levels of RGS2 (black dashed line) comparable with the expression in proximity to mature intratumoural blood vessels. Carbonic anhydrase 9 (CAIX) staining confirmed that the normal epithelium was less hypoxic than adjacent tumour. Further, high RGS2 expression correlated with proliferative areas (white dashed line). However, increased RGS2 expression was also observed in low proliferative, extremely hypoxic areas at the margin of necrosis (Supplementary Fig. S1). Down regulation of RGS2 in response to hypoxia was further evaluated *in vitro*. Data showed that RGS2 protein expression was suppressed under hypoxic conditions in the androgen-sensitive PC cell line LNCaP and androgen-independent LNCaP-19, an effect clearly visible after 72 hours of hypoxic exposure (Fig. 2b). This data was confirmed by CoCl_2_ treatment of an extended experimental model, where the previous cell lines were complemented with the androgen-independent PC cell line, PC-3 and the immortalised normal myofibroblast cell line,WPMY-1 (Fig. 2c).

### High RGS2 levels correlates with poor patient survival and metastasis

**Figure 3:**
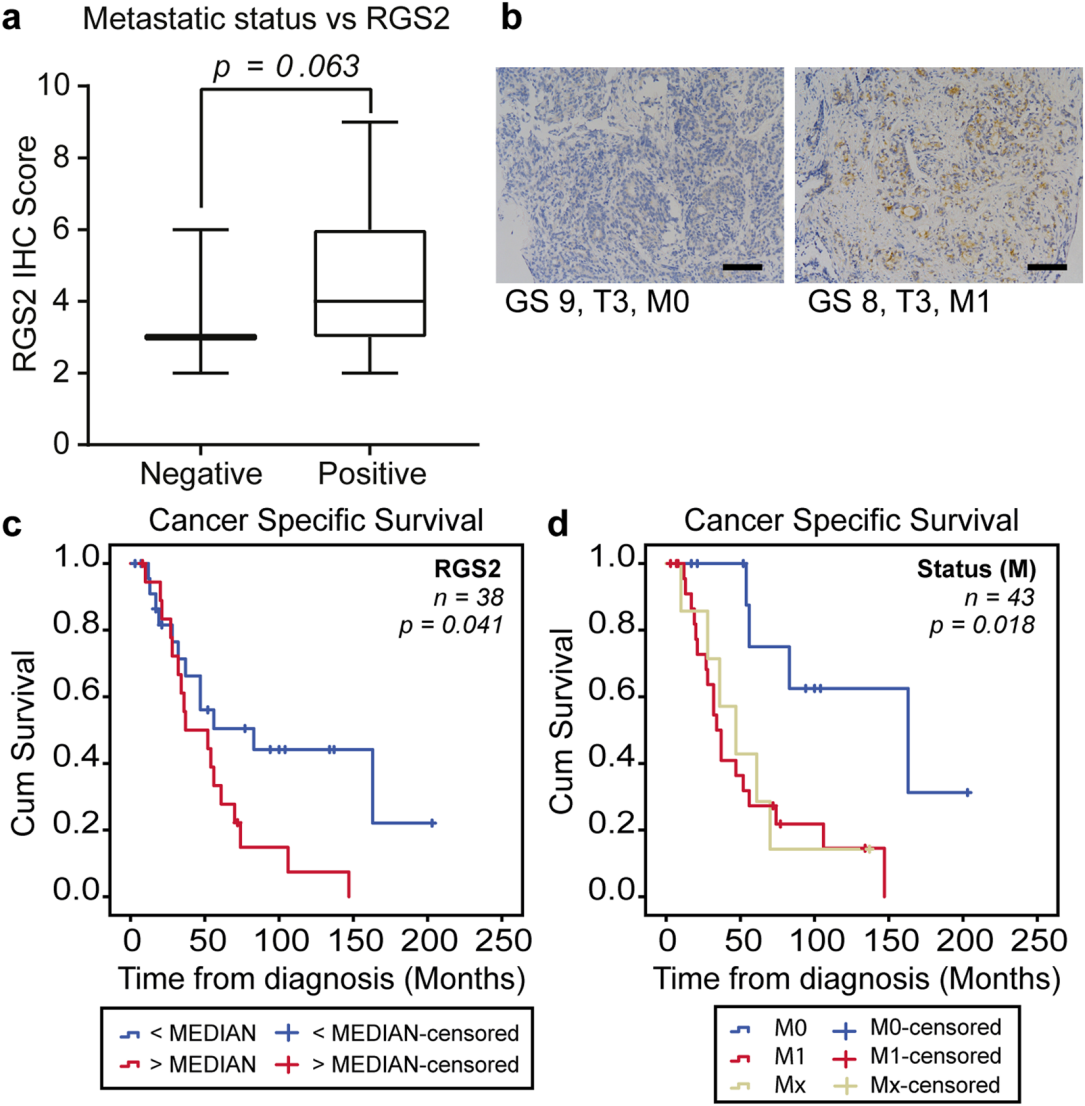
High RGS2 expression is correlated with metastasis and poor prognosis. (**a**) Assessment of RGS2 staining in relation to metastasis in patients with advanced PC. Metastasis positive (*n* = 27) and negative (*n* = 11). (**b**) Representative images of RGS2 staining of matched specimens of M0 and M1. Bar represents 100 µm. Kaplan-Meier curves and log rank of (**c**) cancer specific survival and high respective low RGS2 expression (p = 0.041, by Log-rank test) and (**d**) cancer specific survival and M-stage (*n*_*M1*_ = 24, *n*_*M0*_ = 12, *n*_*Mx*_ = 7, p = 0.018, by Log-rank test).

For assessment of RGS2 expression during tumour progression, a second cohort of advanced PC with higher heterogeneity was IHC stained for RGS2. No correlation was found between RGS2 expression as a continuous variable and the clinicopathological factors tested, except a trend for M-stage (Spearman’s Rho 0.301, p = 0.062). Moreover, analysis of RGS2 score in tertiles showed significant correlation to M-stage (Spearman’s Rho 0.349, p = 0.032) but to no other factors. Furthermore, the RGS2 level was evaluated in relation to metastatic spread (distant and regional lymph node) with indication of an association between high RGS2 and positive metastatic status (Fig. 3a,b).

For evaluation of the prognostic power of RGS2 staining the patients was dichotomized with the median value as cut-off. High RGS2 level at the time of diagnosis was associated with a shortened cancer specific survival (CSS). The mean CSS for patients with low RGS2 was 101.7 months compared to 54.1 months for the patients with high RGS2 expressing tumours (Fig. 3c). For comparison the impact of M-stage was evaluated; the mean CSS was 129.8 months in the M0 group, 55.3 months in the M1 group and 55.6 months in the Mx group (Fig. 3d).

Further, univariable cox analysis confirmed that RGS2 was a prognostic factor for CSS. In fact, RGS2 and M-stage were the only factors with prognostic significance in the current patient cohort (Table 1, univariable analysis). RGS2 was not confirmed significant in a multivariable model with M-stage (Table 1, multivariable model 1). However, when analysed together with PSA, RGS2 was confirmed as an independent prognostic factor that outperformed PSA (Table 1, multivariable model 2). Noteworthy, unadjusted RGS2 had a better prognostic value than the factors used for diagnosis today (Table 1, multivariable model 3).

**Table 1:** Cox regression analysis of clinicopathological factors in PC.

Cohort 2	Univariable analysis		Multivariable model 1	
*(n*_*TOT*_ = *45, n*_*PCD*_ = *29)*			Significant factors	
Variable	HR (95% CI)	p Value	HR (95% CI)	p Value
RGS2 (Cat.)	2.172 (1.008–4.679)	0.048	1.221 (0.505–2.953)	0.658
M-stage (Cat.)	4.223 (1.412–12.632)	0.01	3.911 (1.243–12.308)	0.020
PSA (Log10)	1.860 (0.990–3.493)	0.054		
GS (Cat.)	1.645 (0.279–1.323)	0.210		
T-stage (Cat.)	0.589 (0.139–2.495)	0.472		
Age (Cont.)	0.988 (0.938–1.041)	0.643		
	**Multivariable model 2**		**Multivariable model 3**	
	**Biomarker**		**Current diagnostic factors**	
**Variable**	**HR (95% CI)**	**p Value**	**HR (95% CI)**	**p Value**
RGS2 (Cat.)	2.08 (0.915–4.728)	0.032		
M-stage (Cat.)				
PSA (Log10)	1.555 (0.806–3.001)	0.188	1.873 (0.987–3.552)	0.055
GS (Cat.)			1.786 (0.810–3.938)	0.151
T-stage (Cat.)			0.611 (0.141–2.648)	0.51
Age (Cont.)				

Results from Cox regression analysis presented as hazard ratio (HR) with the 95% confidence interval (CI) and p-value. For RGS2 the median categories were used. GS was divided according GS6-7 vs. GS8-9, T-stage T1-T2 vs. T3-T4, M-stage M0 vs. M1. Age was used as a continuous variable and for PSA a log10 transformation was applied. Additional abbreviation: Prostate cancer death (PCD).

### RGS2 level contribute to the PC phenotype

**Figure 4:**
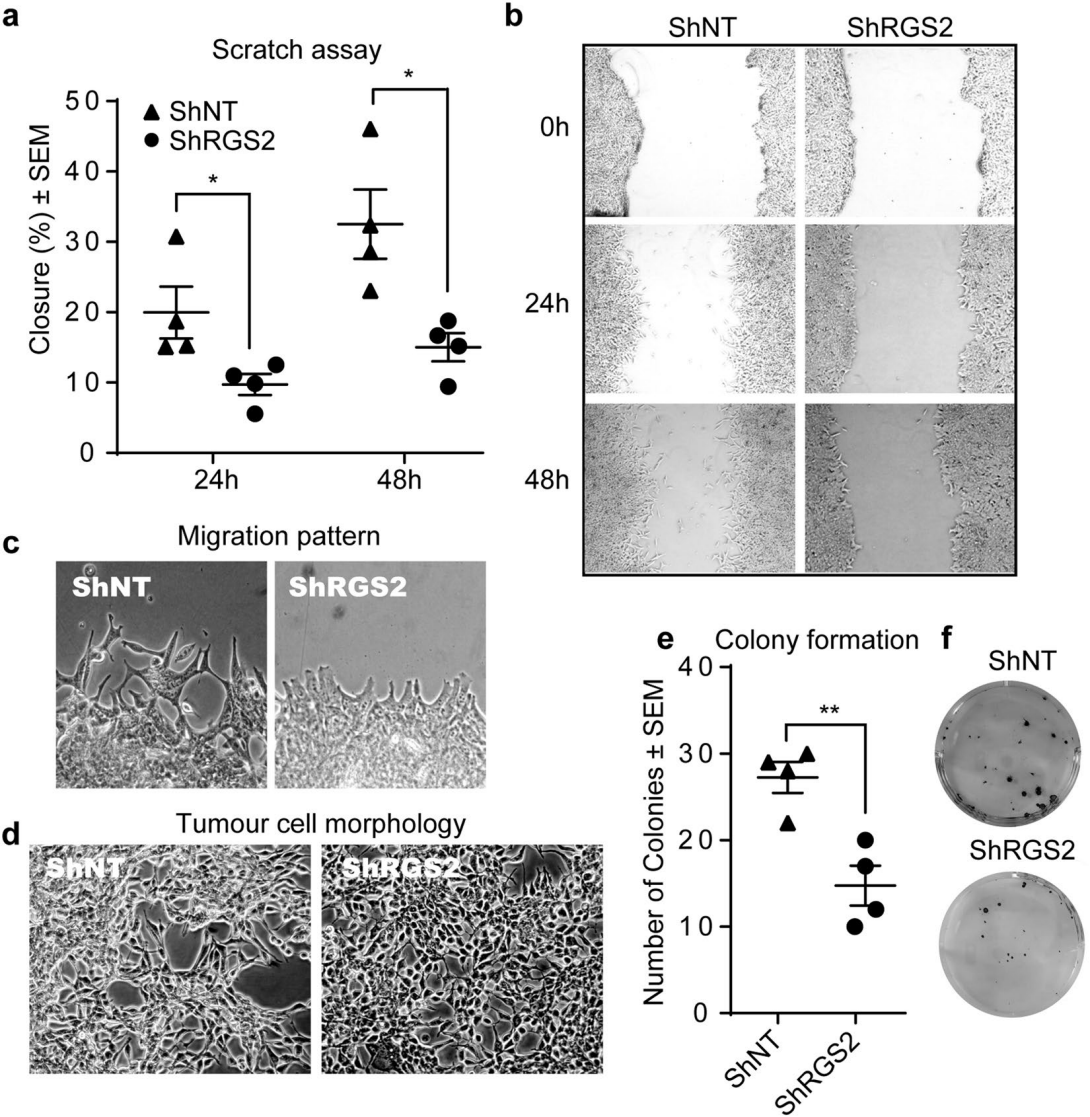
Increased epithelial features in response to RGS2 downregulation. (**a**) Quantitative comparison of migrating properties of the sh-clones, presented as percentage of area of gap closure compared to the starting point. (*n* = 4; 24 h p = 0.042, 48 h p = 0.017, by two-tailed Student’s *t* test). (**b**) Representative images of scratch assay at analysed time points. (**c**) Magnification of the scratch for visualization of migration pattern. (**d**) Phase imaging of the Sh-clones for demonstration of morphology and growth pattern. (**e**) Quantification of clone formation (*n* = 4, p = 0.0051, by two-tailed Student’s *t* test). (**f**) Images of colony formation ability of low seeded clones stained with crystal violet.

In order to evaluate the relationship between RGS2 expression and PC phenotype, RGS2 was knocked-down in the high RGS2 expressing cell line LNCaP. In agreement with the findings from the patient material, high RGS2 expressing PC cells displayed significantly higher metastatic properties than low expressing cells. The impact of RGS2 on tumour cell phenotype was assessed with scratch assay, phase-contrast imaging and colony formation assay. A significant delay in wound closure was detected comparing the low RGS2 expressing cells (ShRGS2) to the control (ShNT) (Fig. 4a,b). Also, a unidirectional migration pattern was evident for the ShRGS2 contrasting the random migration of ShNT (Fig. 4c). Evaluation of morphology showed a more epithelial-like phenotype of ShRGS2, i.e. a more organized growth pattern, increased polygonal shape and consistent dimensions. ShRGS2 also showed decreased tendency to overgrowth compared to ShNT when cultured at the same confluence (Fig. 4d). Additionally, ShRGS2 cells displayed impaired ability to form colonies (Fig. 4e,f). Thus, low RGS2 expression was associated with low metastatic properties while high RGS2 level was associated with basic metastatic traits.

### Decreased RGS2 level is associated with tumour cell survival and declined proliferation

**Figure 5:**
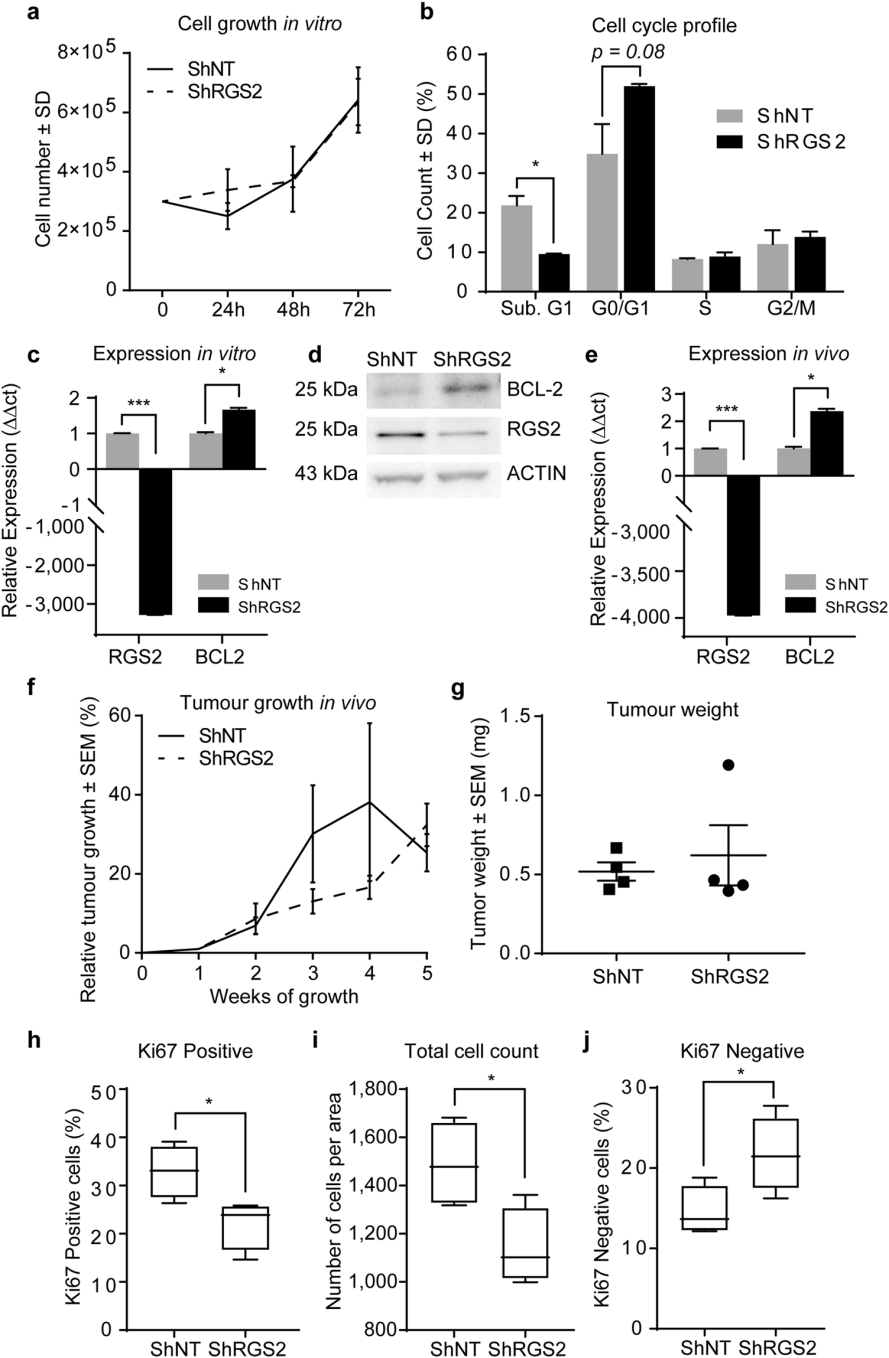
Impact of decreased RGS2 expression on tumour survival and cell growth. (**a**) Total cell growth of the RGS2 knockdown clone (ShRGS2) and corresponding non-target control clone (ShNT) evaluated with trypan blue dye positive cell count. (**b**) Flow cytometric analysis of cell cycle profile of PI-stained ShRGS2 and ShNT cells cultured under standard conditions. (*n* = 3, SubG1 p = 0.019, G0/G1 p = 0.070 student’s *t* test) (**c**) qRT-PCR evaluation of RGS2 and BCL2 mRNA expression *in vitro* (*n* = 3; RGS2 p < 0.00001, BCL-2 p = 0.040, Student’s *t* test). (**d**) RGS2 and BCL-2 protein expression *in vitro* analysed with western blot. The blots were cropped. The original pictures for the evaluation of the clones can be found in Supplementary Fig. S42. (**e**) RGS2 and BCL-2 gene expression in subcutaneous tumours after implantation of ShRGS2 (*n* = 4) and ShNT (*n* = 4) evaluated with qRT-PCR (*n* = 4; RGS2 p = <0.00001, p = 0.0168, student’s *t* test). (**f**) Relative tumour growth *in vivo* (*n* = 4) and ShNT (*n* = 4) in BALB/c nude mice. **(g)** Tumour weight at experiment endpoint. (**h**) Comparison of Ki67 positive cells (p = 0.0277, student’s *t* test). (**i**) Comparison of total cell count per hot spots (p = 0.028, student’s *t* test). (**j**) Assessment of Ki67 negative cells (p = 0.045, student’s *t* test).

Further evaluation of RGS2 and PC phenotype showed that although ShRGS2 displayed similar growth rate when compared to ShNT (Fig. 5a), cell cycle analysis revealed that ShRGS2 cells were less proliferative, shown by an increased cell number with diploid DNA content (G0/G1). Moreover, increased survival of shRGS2 cells was shown by reduction of the apoptotic population, Sub-G1 (Fig. 5b). Moreover, RGS2 knockdown resulted in increased gene expression of the anti-apoptotic BCL-2 (Fig. 5c) and corresponding protein expression (Fig. 5d). In accordance, increased level of BCL-2 gene expression in response to RGS2 reduction was also demonstrated *in vivo* (Fig. 5e). Assessment of tumour cell growth *in vivo* showed that tumour establishment was similar between the two clones. An initial growth advantage of RGS2 expressing tumours (ShNT) was seen (Fig. 5f). However, a gradual decline entailed overlap of mean tumour size between the two groups at the time of experiment termination (Fig. 5g) Tumours from both groups displayed tissue haemorrhage associated with reoccurring visible haematomas that correlated with a major decrease in tumour volume. The ShRGS2 tumours however displayed significantly less tissue haemorrhage hence less tumour volume variance (see Supplementary Fig. S2). Ki67 staining for assessment of proliferation confirmed that ShRGS2 were less proliferative than the control (Fig. 5h). In agreement, the decreased total cell count confirmed this data (Fig. 5i). Furthermore, significant increase of the Ki67-negative cell population of ShRGS2 (Fig. 5j) corresponded with the decreased G0/G1 cell count observed *in vitro*.

### RGS2 expression level has distinct effect on signalling pathways associated with PC progression

**Figure 6:**
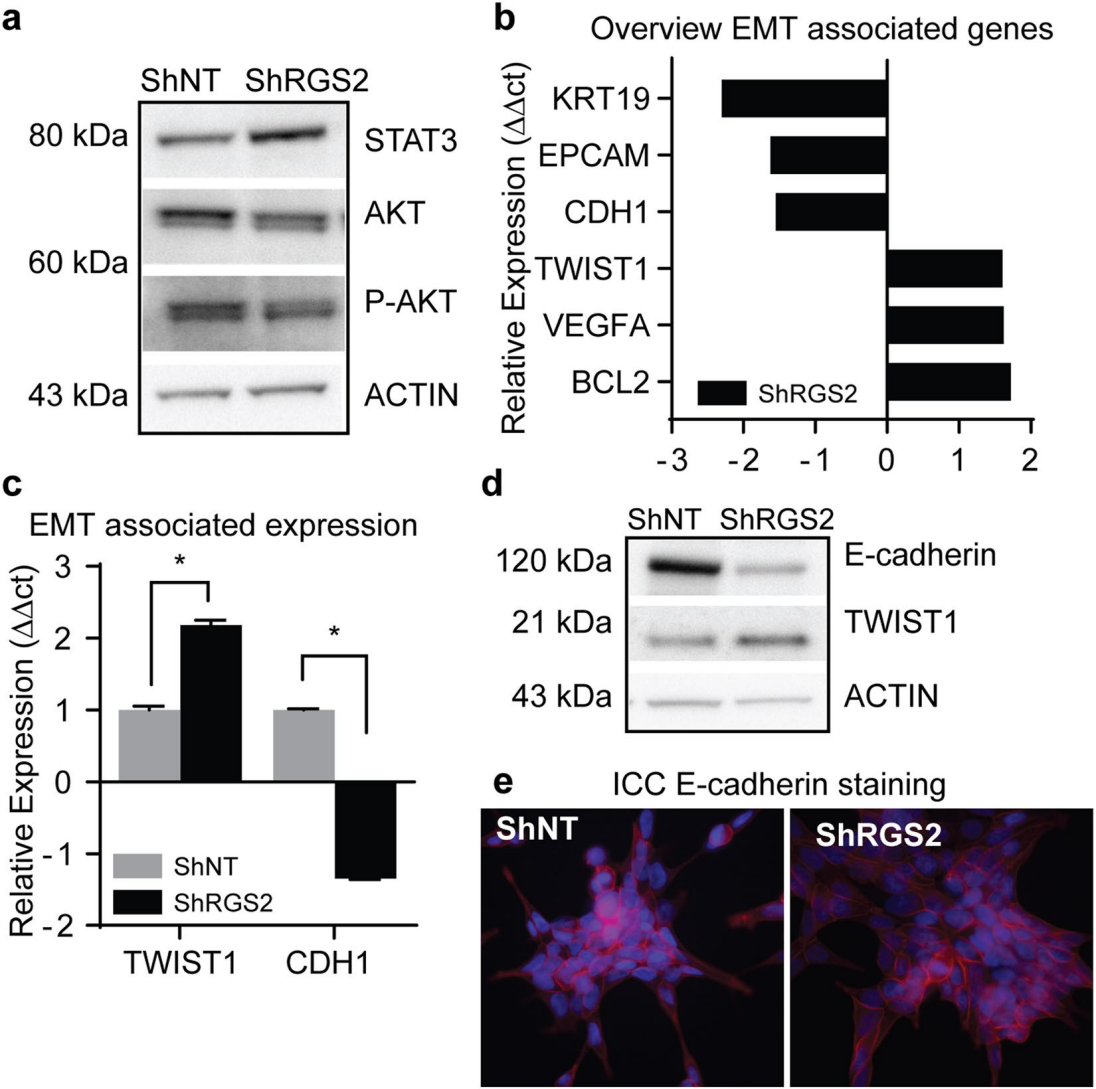
Affected Pathways and EMT initiation in response to RGS2 depletion. (**a**) Evaluation of STAT3 and AKT proteins in response to RGS2 knockdown using WB. (**b**) Overview of evaluated EMT markers from a PC gene expression panel (this data represent a single replicate). (**c**) qRT-PCR quantification of mRNA expression of EMT associated TWIST1 and E-cadherin of TWIST1 and CDH1 (*n* = 3; TWIST1 p = 0.021, CDH1 p = 0.034, student’s *t* test). (**d**) Representative western blots for corresponding protein expression. The blots were cropped and gels were run at the same conditions. The original pictures for the evaluation of the clones can be found in Supplementary Fig. S4). (**e**) Immunocytochemistry **(**ICC) analysis of localization of E-cadherin expression (red) and nuclei counterstained with DAPI (blue).

The PI3K/AKT pathway was downregulated in response to RGS2 knockdown, shown by decreased levels of total-AKT (besides p-AKT) in ShRGS2 compared to ShNT. Moreover, overall STAT3 protein expression was upregulated in shRGS2 (Fig. 6a).

Further, downstream effects of decreased RGS2 expression were addressed using a panel of 47 PC-markers, 17 entries showed a relative expression difference of 1.5 or more between ShRGS2 and ShNT. Six out of seven EMT associated genes showed a relative expression corresponding to an EMT profile, i.e. upregulation of TWIST1, BCL-2 and VEGFA and complementary downregulation of EPCM, CDH1, KRT19 in ShRGS2 cells (Fig. 6b). The expression of TWIST1 and CDH1/E-cadherin was confirmed with single qRT-PCR and WB, and both gene and protein expression were consistent with results from the panel (Fig. 6c,d). Additional EMT markers were evaluated using single qRT-PCR but showed equal expression comparing the two clones (SNAI1, SNAI2, VIM, CDH2, CD44 and ZEB1).

Evaluation of cellular localization of E-cadherin did not show any obvious alterations in protein levels. However, although both cell lines displayed general membrane associated E-cadherin, the ShRGS2 populations displayed a patchy expression pattern with less stringent intensity and localization (Fig. 6e).

## Discussion

The understanding of PC development and progression has constantly increased, and during recent years several new therapies have entered clinical practice, mostly in the CRPC phase, but also in earlier stages of the disease. Despite these advances, the tools of today are not sufficient to accurately characterize the disease and optimize or individualize treatment. Thus, further elucidation of mechanisms involved in PC progression and putative future biomarkers or drug targets are needed.

Decreased RGS2 expression in PC has previously been reported^15^, however, the cause and phenotypic alterations in the hormone-naïve state have not been described. In this study, we aimed to scrutinize aberrant RGS2 expression in terms of regulation and tumour advantage but also evaluate potential for diagnostic purposes and treatment.

In line with a previous study^15^, RGS2 was found down regulated in early low grade PC compared to benign tissue. Aberrant RGS2 expression has been described in response to hypoxia in several cell- and tissue types and seems to be context and tissue dependent^19,22,23^. The present study describes high RGS2 expression in blood vessel proximity and progressively decreased expression towards increasingly hypoxic tumour regions. The approximate ≤100 µm oxygen diffusion limit was mirrored by the RGS2 expression pattern. Further evaluation of this observation showed that RGS2 expression was inhibited by hypoxia and HIF1 in all the prostate originating cell lines tested. This suggest that downregulation of RGS2 is a general mechanism, shown by corresponding results subordinate the characteristics of the different cell lines. This is also in line with the correlation between epithelial and stromal RGS2 staining observed in the patient material. Additionally, the data also show that although normal epithelia in the patient specimens expressed marginally more RGS2 than adjacent cancer cells, these regions displayed significantly less RGS2 staining than BPH specimens from cancer free patients (median score 2 and 8 respectively). This is in accordance with described hypoxia also in normal areas adjacent tumours^49^. However, an elevated level of RGS2 expression in advanced PC suggests that the tumour cells overcome the initial suppression. Additionally, several studies has shown that advanced PCs are more hypoxic than low grade^49–51^ and though PC is considered a generally hypoxic cancer, necrosis has been shown limited to high Gleason score GS (≥9)^52^. The upregulation of RGS2 in exceedingly hypoxic areas in the animal model suggests that severe hypoxia could induce RGS2 in advanced PC. This is in agreement with induction of RGS2 expression in response to various stress^9,22,23,53^. Altogether, these data suggest a dose dependent hypoxia regulation of RGS2 expression in PC.

The present study shows a correlation between high RGS2 expression and poor prognosis, a novel observation for PC. The corresponding results from the correlation of M-stage and CSS, together with the association between high RGS2 staining and a positive metastatic status suggest that RGS2 expression is associated with PC metastasis. The lack of correlation between GS or T-stage and RGS2 score supports the association to metastasis rather than local progression without tumour dissemination. However, although the results propose that the prognostic value of RGS2 is at large reflecting the metastatic status, data also suggest that stratification of patients into high and low risk groups can be based on RGS2 staining with good accuracy at an early time point. This statement is also supported by the Cox regression analysis showing that RGS2 has an independent prognostic value. Numerous reports from different types of cancer linking increased RGS2 expression to diverse cancer phenotypes and patient outcomes^16–20^ suggest that tumour properties and origin are influential. These findings need further evaluation, but points towards distinct roles for RGS2 during PC progression and suggest a prognostic value by itself or together with PSA. Importantly, opposing previous suggestion of RGS2 protein stabilization as a treatment target for advanced PC^54^; our data advice that such treatment could have hazardous effects considering metastatic properties in association to high RGS2 levels.

In agreement with the patient data, we could show that cancer cells expressing low RGS2 levels displayed decreased metastatic properties compared to high expressing cells in terms of motility and colonializing ability. In line, the morphology of ShRGS2 was more epithelial-like. Unidirectional movement and growth pattern suggested increased cellular polarity consistent with traits of a less dedifferentiated tumour cell phenotype. Furthermore, our data show that RGS2 knockdown had a significant effect on cell survival and an equivalent opposing influence on proliferation. In line with the decreased apoptosis of ShRGS2, increased expression of the anti-apoptotic BCL-2 was detected. The increased BCL-2 expression was sustained *in vivo* endorsing the relevance of decreased RGS2 expression. This suggests a survival advantage of low RGS2 expressing tumour cells by the BCL-2 associated prevention of hypoxia-induced cell-death^26,27,29^. Additionally, in line with the common slow growing phenotype of primary PC, shRGS2 was significantly less proliferative than the RGS2 expressing clone.

Evaluation of signalling pathways affected by RGS2 knockdown reveal increased expression of STAT3. Although RGS2 has been reported to repress STAT3 activity^55^, this is the first report of RGS2 associated effects on STAT3 expression. STAT3 is known to regulate both cell survival via BCL-2^56,57^ and cancer progression via e.g. TWIST1^58^. Consistent with reports of hypoxic stimulation of STAT3^59,60^ this observation links not only decreased RGS2 expression to hypoxia induced cell survival but proposes a mechanism behind the EMT-like expression profile of ShRGS2 compared to ShNT. This suggests that downregulation of RGS2 is not only a consequence of EMT but a contributing factor. Though suppressed E-cadherin by key transcription factors such as TWIST1 is considered a hallmark of EMT^61,62^ the incomplete EMT transcription profile and ambiguous E-cadherin expression suggest that additional signals are needed for further tumour cell dedifferentiation.

Despite the fact that STAT3 activation has been associated with PC progression and EMT^42,44,63^, inhibition of STAT3 activity has failed as treatment for metastatic PC^40,64,65^. In fact, it has been suggested that for PC with loss of Phosphatase and tensin homolog (PTEN) and constitutively active PI3K/AKT pathway, represented here by LNCaP, STAT3 inactivation induce metastasis^40^. Furthermore, the complementary downregulation and inactivation of the PI3K/AKT pathway in response to RGS2 knockdown put forward a potential mechanism linking high RGS2 to later dedifferentiation and metastasis. In summary, this reflects a stepwise progression where RGS2 may have a regulatory role by shifting the emphasis between the STAT3 and PI3K/AKT pathways. If the regulatory action of RGS2 is carried out by inhibition of translation^8,9^, stimulation of transcription^10^ or inhibition of G- protein signalling^11–13^ is not answered here but the data suggests that RGS2 is involved in mechanisms central for PC progression.

In conclusion, we suggest that RGS2 levels contribute to the PC tumour cell phenotype. Low RGS2 expression is associated with an indolent cancer phenotype representative of a major fraction of primary PC. On the other hand, high RGS2 correlates with a proliferative metastatic phenotype associated with progressed cancer. Hence, we propose that high RGS2 contribute to the aggressive phenotype of advanced PC. Clinically, our data suggests that RGS2 expression has a prognostic value and the potential to distinguishing between low- and high-risk patients. However, this finding needs to be confirmed by a prospective study. Additionally, we suggest that the downregulation of RGS2 seen in early stages of PC is caused by moderate hypoxia and suggests that decreased RGS2 expression contribute to improved tumour cell survival and priming for later EMT. Finally, RGS2 expression could be indicative of therapeutic opportunities with distinctive expression pointing towards treatment windows for the STAT3 and PI3K/AKT signalling pathways.

## Methods

### Patient material

*Cohort I*. Archival material consisting of 36 benign prostatic hyperplasia (BPH) and 28 untreated (hormone-naïve (HN)) prostate cancer specimens in stage T1b (more than 5% adenocarcinoma within the tissue resected) was analysed immunohistochemically (IHC) for RGS2 expression. Specimens were obtained by transurethral resection of the prostate (TURP). The patients included in the study were between 60 and 90 years old. Tumours were morphologically classified according to Gleason. Mean Gleason score was 6.8. Additionally, at time of diagnosis M-stage and PSA level were known for the PC patients.

*Cohort II*. In addition, 45 tissue specimens ranging from T1c to T4 were analysed for RGS2 expression. The samples where obtained by needle biopsy at the time of diagnosis prior to androgen depravation therapy (ADT). The included patients were between 54 and 87 years old. Mean Gleason scores in the group was 7.3. The patients included in the study were diagnosed at the Department of Urology, Sahlgrenska University Hospital, Gothenburg, Sweden. Information about Gleason score (GS), M-stage, age, T-stage and PSA level were available for the majority of the patients included in the study. Association between RGS2 expression and known clinicopathological factors were evaluated. Date and cause of death were assessed for each patient. For specification of patient data of the two included patient cohorts see Supplementary Table S1.

Procedures and use of anonymised material, remains from diagnostic care, were performed according to ethical guidelines. Informed consent was obtained from all patients included in the study. The study was approved by the local ethical committee at the Sahlgrenska University Hospital in Gothenburg (reference number cohort I: 11608 and Cohort II: 667-05).

### Immunohistochemistry

Human archival and animal specimens were deparaffinised and rehydrated and endogenous peroxidase was inhibited with 3% H_2_O_2_ in methanol before IHC. RGS2 (0.8 µg/ml, ab36561, AbCam, Cambridge, UK) was detected using the Vectastain ABC kit (Vector Laboratories, Inc., Burlingame, CA). Negative controls included were human RGS2 blocking-peptide (5 µg/ml, ab36560, AbCam), rabbit IgG (0.8 µg/ml, Normal rabbit IgG control, R&D Systems, Abingdon, UK) and excluded primary antibody. Primary antibody incubation was carried out over night at 4 °C. Antibody binding was visualized by DAB+ (Dako, Agilent technologies, DK) and counterstained with Mayer’s hematoxylin. RGS2 protein expression was scored blinded. The *RGS2 score* was calculated by multiplication of the intensity and the percentage of the epithelial cells stained for RGS2. The intensity was scored as: 0 = no detectable staining, 1 = weak staining, 2 = moderate staining, and 3 = strong staining; the proportion of positive cells was scored as 0 < 5%, 1 ≤ 33%, 2 ≤ 66%, and 3 > 66% positive cells. The median expression per group was calculated generating a RGS2 score. For assessment of RGS2 expression in prostate epithelial cells, BPH and PC (both cohorts) the entire specimen was evaluated. Stromal staining was evaluated for three high RGS2 expressing areas (aka hotspots) per specimen and compared to the epithelial staining of the same area. Tumour adjacent stroma in view at 10 times (10X) magnification (surrounding and interspersed) where assessed and calculated alike.

Anti-Ki67 (MA5-14520, Thermo Fisher Scientific, Rockford, IL) was used at a 1:400 dilution. Cells were counted at 20 times (20X) magnification for two hotspots per tumour. Staining was classified according to: positive = strong staining, negative = no staining and ambiguous = weak staining. Cell count data was obtained with the ImageJ software. Anti-CD31 (77699, Cell signaling, Leiden, NL) was used at a 1:200 dilution. Evaluation of vessel area was analysed with the BioPix iQ 3.3.1 software. Anti-CAIX (ab15086, Abcam) was used at a 1:1500 dilution. IHC was performed as described above. Anti-HIF1A (ab51608, Abcam) was used at a 1:100 dilution and incubated for 1 h at room temperature.

Hematoxylin and eosin staining was carried out according to standard protocol. Staining was evaluated with the BioPix iQ 3.3.1 software. Large necrotic areas were omitted from the analysis for evaluation of tissue haemorrhage.

### Cell culture

Cells were cultured at 37 °C in humidified air with 5% CO_2_ under atmospheric oxygen pressure unless stated differently. LNCaP (ATCC® CRL-1740™, Manassas, VA) and PC-3 (ECCC, Wiltshire, UK), 22Rv1 (ATCC® CRL-2505™) and LNCaP-19 (castration-resistant LNCaP sub-line, characterized elsewhere)^66^ were cultured in RPMI-1640 (PAA Laboratories, Pasching, Austria) containing stable glutamine, supplemented with 1 mM sodium pyruvate and 10% fetal bovine serum (FBS; Gibco, South America) or 10% Dextran-Charcoal Stripped FBS (DCC; Gibco, South America), for LNCaP-19. The myofibroblast cell line WPMY-1 (ATCC® CRL-2854™) and VCaP (ATCC® CRL-2876™) was cultured in Dulbecco’s Modified Eagle’s Medium supplemented with 5% and 10% FBS respectively. Sh-clones were cultured according to protocol for LNCaP. All cells were cultured with antibiotics (Penicillin-Streptomycin; Thermo Fisher Scientific) and routinely tested for mycoplasma contamination. *In vitro* experiments were conducted in biological triplicates if not stated differently.

### Hypoxia

Hypoxia response was evoked by exposure to reduced oxygen levels (1% O_2_) in a hypoxia chamber (Sci-tive-N hypoxia workstation, Ruskinn Technology, Bridgend, UK) and control cells were cultured in atmospheric oxygen. Samples were taken after 48 and 72 hours. The hypoxia chamber was manually calibrated, and oxygen levels were verified frequently. Following hypoxia exposure, cells were kept on ice, rinsed with cold D-PBS (PAA Laboratories) and lysed directly in the culture flask to reduce HIF1Α protein degradation. Complementary experiments with CoCl_2_ (Sigma-Aldrich, St Louis, MO) were performed to validate the hypoxia data. CoCl_2_ treatment (150 µM) was carried out under normal atmospheric oxygen for 48 or 72 hours.

### Western blot

Cell pellets for protein analysis were lysed on ice with CelLytic™ M cell lysis reagent (Sigma-Aldrich) with the addition of phosphatase inhibitors (PhosSTOP; Roche, Applied Science, Penzberg, Germany) and protease inhibitors (cOmplete, Mini, Roche). Following sonication the samples were cleared of debris by centrifugation. Proteins was separated on SDS-PAGE gels (Novex, NuPAGE 4–12% Bis-Tris Gels; Invitrogen, Carlsbad, CA) and transferred to PVDF membranes (iblot® transfer stack, Invitrogen). Gels were stained with SimplyBlue™ Safe stain (Invitrogen) and membranes were stained with Ponceau S solution (Sigma-Aldrich), to verify equal loading and transfer. Membranes were blocked with 5% BSA, (Albumin from bovine serum; Sigma-Aldrich) TBS-Tween (0.15 M NaCl, 0.05 M Tris-HCl, pH 7.6 and 0.1% Tween-20). All washings were carried out with TBS-Tween, and all antibodies were diluted in 2% Amersham™ ECL Prime Blocking Agent (GE Healthcare, Little Chalfont Buckinghamshire, UK). Antibody binding was visualized by enhanced chemiluminescence using Amersham™ ECL select Western Blotting Detection Reagent (GE Healthcare) and the LAS1000 image- detection system (Fujifilm Life Science, Stamford, CT). The following antibodies were used: anti-RGS2 (H00005997-M0; Abnova, Taipei, Taiwan), anti-RGS2 (ab36561; AbCam), anti-TWIST (Sc-81417; Santa Cruz Biotechnology, Dallas, TX), anti-BCl-2 (ms-123-p1; NeoMarker, Fremont, CA), anti-E-Cadherin (610182; BD Transduction laboratories; Franklin Lakes, NJ), anti-β actin (A5441; Sigma-Aldrich), anti-Pan-AKT (4691; Cell signaling), anti-p-AKT (4058; Cell signaling), anti-STAT3 (12640; Cell signaling).

### Knockdown of human RGS2 in LNCaP

SureSilencing RGS2 shRNA Plasmid was purchased from Qiagen (KH02231N, Venlo, Qiagen). Transfection with four different Short hairpin RNA (ShRNA) constructs for RGS2 silencing together with non-target (NT) shRNA for control was performed. The stably transfected cells were selected with Gentamicin (GS14), clones were picked using a sterile pipette-tip, expanded and analysed for RGS2 knockdown efficiency. The clone with less RGS2 expression was chosen for further analysis. The control clone was compared to the mother cell line LNCaP and showed to be similar if not identical in terms of RGS2 expression, cell cycle profile, growth rate and general expression profile. The tumour phenotype of subcutaneous mouse tumours also compared well to LNCaP originating tumours.

### Analysis of cell cycle and apoptosis

Cells were detached from the culture dish and collected together with the original culture media. After centrifugation and D-PBS wash of the pellet cells were resuspended and incubated in Vindelov’s reagent (75 μmol/L PI, 20 mmol/L Tris, 100 mmol/L NaCl, 0.1% Nonidet p-40, and 20 µg/ml RNase adjusted to pH 8.0) at a cell concentration of ≤10^6^ cells per ml. Staining were analysed with a FACSCalibur flow cytometer (BD Biosciences) using the FL3 channel in linear scale for cell cycle distribution. Cells were considered apoptotic when showing less than diploid DNA content (Sub-G1) in a logarithmic FL2 channel.

### Quantitative real-time PCR

Tumour tissue was homogenized using a TissueLyser (Qiagen, Hilden, Germany). Cell pellets were homogenized by reflux through a 20 gauge syringe. RNA was isolated using the RNeasy Mini Kit (Qiagen) according to protocol recommended by the manufacturer. Reverse transcription was performed for 1 µg RNA using the SuperScript® VILO™ cDNA Synthesis Kit (Invitrogen). Quantitative real-time PCR was carried out using TaqMan® Fast universal PCR system (Applied Biosystems™, Foster City, CA) with the following gene specific TaqMan® Gene Expression Assays (Applied Biosystems™, Foster City, CA): *RGS2* Hs00180054_m1, *TWIST* Hs00361186_m1, *CDH1* 01023894_m1, *BCL2* Hs00608023_m1, *VIM* Hs0095811_m1, *CDH2* Hs0098056_m1, *SNAI1* Hs00195591_m1, *ZEB1* Hs00232783_m1, *CD44* Hs01075861_m1, *GAPDH* Hs02758991, *HIF1Α* Hs00153153_m1, *CASC3* Hs00201226_m1, *GUSB* Hs00939627_m1. Ct values were defined by ABI 7500 Real-TimePCR System and expression was calculated using the delta-delta method. For *in vivo* analyses the expression was calculated relative the endogenous control human GAPDH or GUSB, CASC3 was used for *in vitro* experiments.

For a gross overview of potential down-stream expressional effects of RGS2-knockdown a TATAA GrandPerformance Assay Panel^67^ and ValidPrime™ assay was performed for evaluation of altered expression of the 47 included genes. qPCR was performed on BioMark (Fluidigm) using the 96.96 Dynamic Array™ IFC (Integrated Fluidic Circuit). Reverse transcription pre-amplification and qPCR was performed at TATAA Biocenter (Gothenburg, Sweden). Samples were analysed in one replicate.

### Animal experiment

A total of 10 male BALB/c nude mice (Charles River Laboratories International, Inc., Wilmington, MA), was used in the experiment. Two million cells, resuspended in 200 µl of 1:1 antibiotic-free media and Matrigel^TM^ (BD Biosciences, Bedford, MA), were injected subcutaneously on the left flank. Animals were sacrificed 10 weeks following implantation or when tumours reached ethically approved tumour volume of 1300 mm^3^. Animals were monitored weekly when also tumours were measured using a calliper. Tumour volume (V) was calculated using the formula V = (length × width^2^)/2. Tumour growth was normalised by calculation of volume relative to first detected tumour volume. Orthotopic implantation in the dorsal-lateral lobe of the prostate was carried out via a T-incision in the lower abdomen. 1 million LNCaP cells in 7 µl of BD Matrigel (BD Bioscience) were implanted using a 30 gauge needle. Harvested tissue was preserved in RNAlater® Solution (Ambion, Austin, TX) for later RNA extraction or fixed in formalin for paraffin embedding. All animal work was carried out according to Swedish ethical rules and regulations. The use of animals and protocol were approved by the regional animal ethics committee in Gothenburg (reference number: 173-2014).

### Scratch assay

Cells was plated onto 6-well plates and cultured to ≥80% confluence. A scratch was made using a sterile pipette tip, culture medium was changed and position markings were made for consecutive imaging following the next 24, 48 and 72 hours. Images were captured using a Nikon TMS inverted microscope equipped with a Lumenera Infinity 1 camera. The gap area was measured using the Infinity software and closure was calculated as area decrease compared to the starting time point.

### Clonogenic assay

10 000 cells were plated onto BioCoat™ Poly-D-Lysine coated 6-Well plates (Corning, NY) and cultured for 21 days. Media was replaced every second or third day. Formed colonies was washed, fixed with 4% PFA and stained with crystal violet (0.5% in 4% PFA) and counted using an invert microscope. Colonies of more than approximately 30 cells were counted.

### Immunocytochemistry

Cells were cultured on 8-well glass slide culture chambers until 70% confluence, fixed with 4% neutral phosphate buffered formalin (4% PFA) on ice for 45 min, washed and stored in PBS until staining. Cells were stained with mouse anti-E-cadherin antibody (610182; BD Transduction laboratories; Franklin Lakes, NJ) for 1 hour in room temperature and incubated with Alexa Fluor^TM^ Plus 555 goat anti-mouse IgG (H+L) secondary antibody (A32727; Thermo Fisher Scientific) in a dark humid atmosphere. Slides were mounted with ProLong™ Diamond Antifade Mountant with DAPI (Thermo Fisher Scientific). Images where captured with a Zeiss Axioskop 2 plus microscope equipped with a Nikon DS-Fi1 camera.

### Statistics

Statistical analyses were carried out using IBM SPSS Statistics, version 20. For all tests *p ≤ 0.05, **p ≤ 0.01, ***p ≤ 0.001 were considered significant. Mann-Whitney U test was performed for evaluation of differences in RGS2 staining between unpaired groups and for evaluation of tissue haemorrhage. Wilcoxon W paired test was used for analysis of intra-sectional differences between normal and malignant areas. Spearman correlation was used for assessment of epithelial and stromal RGS2 staining as well as correlation between different clinicopathological factors. A two-tailed Student’s t-test was used to assess differences in gene expression, scratch and clonogenic assay and for evaluation of proliferation *in vivo*. For survival analysis, a Kaplan-Meier chart was created and Log-rank (Mantel-Cox) was used to define statistical differences. Univariable Cox regression was used to evaluate the association between survival time and known clinicopathological factors. A log10 transformation was applied for variables with a significantly skewed distribution (p-value < 0.01 with Shapiro-Wilk test of normality). When the condition for normality was still not met the median was used as cutoff. Potential interaction between each variable and time was evaluated. Proportion hazard assumption (PH) was not violated or any of the variables.

## Electronic supplementary material

Supplementary Table and figures

## Data Availability

Data generated and/or analysed in this study are included in the article or available from the corresponding author on reasonable request.
