# Supplementary material for: Analysis of regulator of G-protein signalling 2 (RGS2) expression and function during prostate cancer progression
Source: Sci Rep. 2018 Nov 22;8:17259. doi: 10.1038/s41598-018-35332-4 (PMC6250724; doi:10.1038/s41598-018-35332-4)
Supplement: Supplementary file 1 — Supplementary Table and figures [file 41598_2018_35332_MOESM1_ESM.pdf]

Supplementary Information

Analysis of regulator of G-protein signalling 2 (RGS2) expression and function during prostate cancer progression

*Anna Linder[1], anna.linder@gu.se
Malin Hagberg Thulin[2], malin.hagberg.thulin@umu.se
Jan-Erik Damber[1], jan-erik.damber@urology.gu.se
Karin Welén[1], karin.welen@urology.gu.se

[1]Department of Urology, Sahlgrenska Cancer Center, Institute of Clinical Sciences, Sahlgrenska Academy at the University of Gothenburg, Göteborg, Sweden
[2]Department of Radiation Sciences, Division of Oncology, Umeå University, Umeå, Sweden

Supplementary table S1

Patient information of the two cohorts of patients included in this study

**Cohort I *(n = 28)***		
**Clinicopathological factors**	**Range**	***Median***
Age (y)	60 - 90	78
PSA (pre.op)	normal - 200	13.5
Gleason score	5 - 8	6.8
M1	n = 5	
N1	n = 0	
**Cohort II *(n = 45)***		
**Clinicopathological factors**	**Range**	***Median***
Age (y)	54 - 86	76
PSA (pre.op)	12 - 2900	208
Gleason score	6-9	7
M1	n = 26	
N1	n = 1	
T-Stage	T1c - T4	
Treatment: GnRH, TAB, Cast.		

Clinicopathological factors evaluated in relation to RGS2 expression in included patient cohorts. Additional abbreviations: Preoperative (pre.op)

Supplementary figure S1 *RGS2are up regulated in proximity to blood vessels and exceedingly hypoxic areas.*

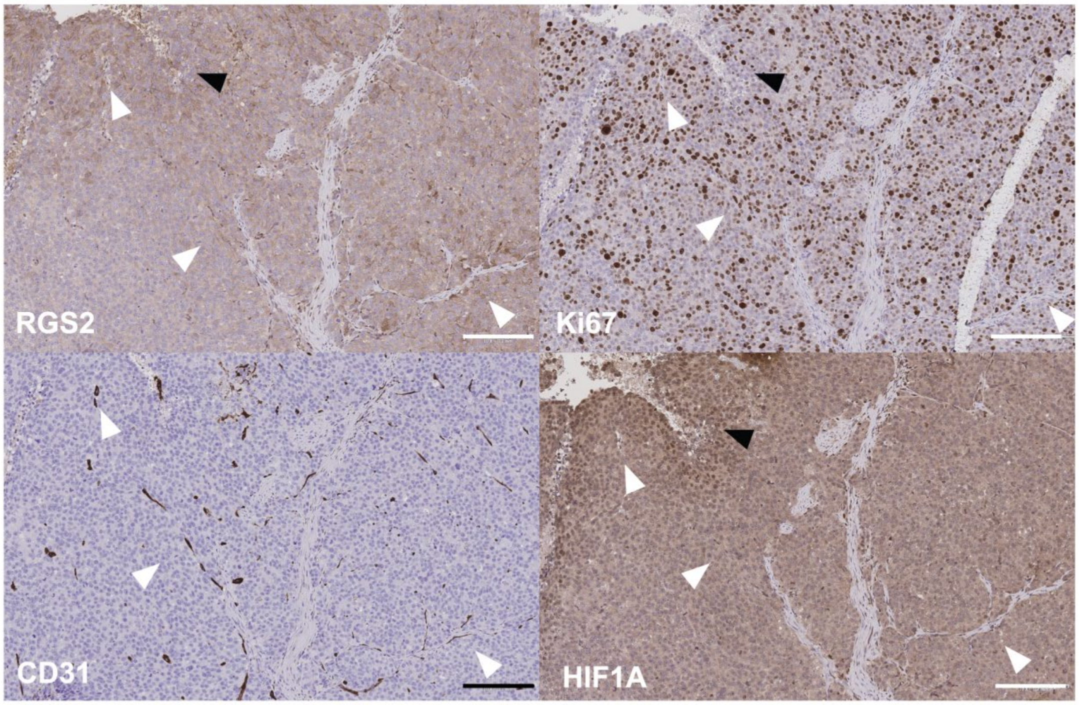

Supplementary figure S1 ***Overview of RGS2 expression in LNCaP descending orthotopic mouse tumours in relation to blood vessels (CD31), proliferation (Ki67) and hypoxia (HIF1A)*** High RGS2 expression in vascularized (white arrowhead) and necrotic areas (black arrowhead)**.** Bar represents 200µm.

Supplementary figure S2 *Evaluation of morphology of subcutaneous tumours*

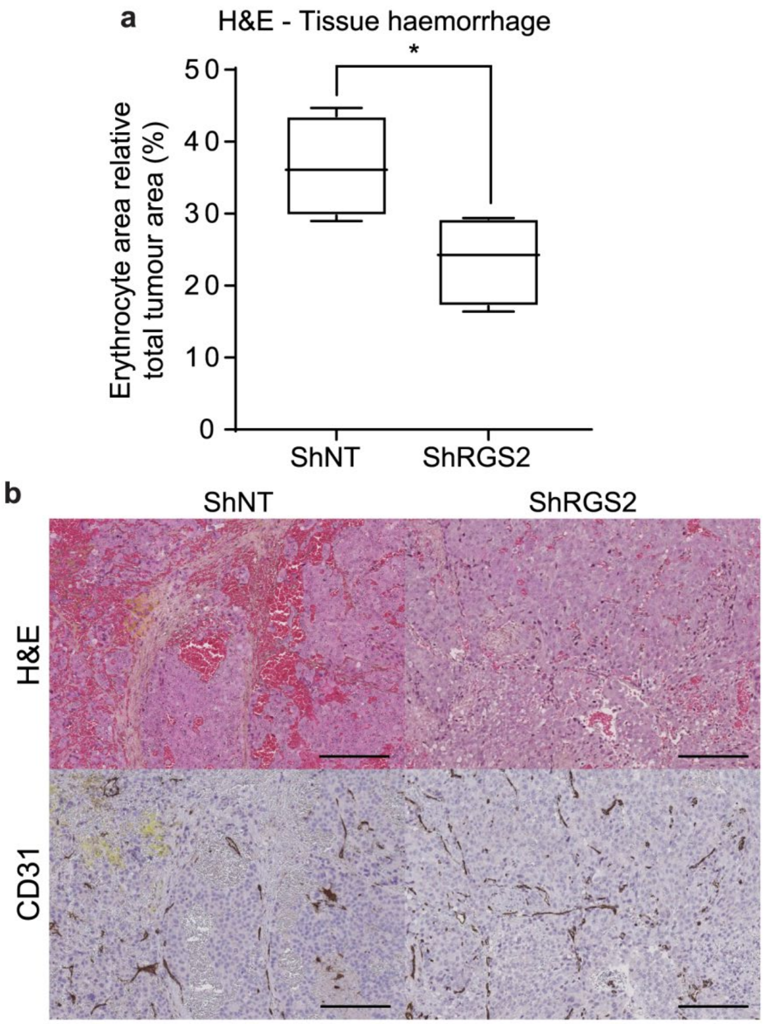

Figure S2 ***Subcutaneous tumours displayed severe tissue haemorrhage.***
a Comparison of tissue haemorrhage area evaluated with ImageJ. ShRGS2 tumours showed significantly less tissue haematoma compared to ShNT (*n = 4*; p = 0.0286, Mann-Whitney test).
b Representative images of H&E stained shNT and shRGS2 originating tumour areas with similar vessel density annotated with CD31 staining. Bar represents 200µm.

Supplementary figure S3 *Hypoxia and $CoCl_2$ treatment*

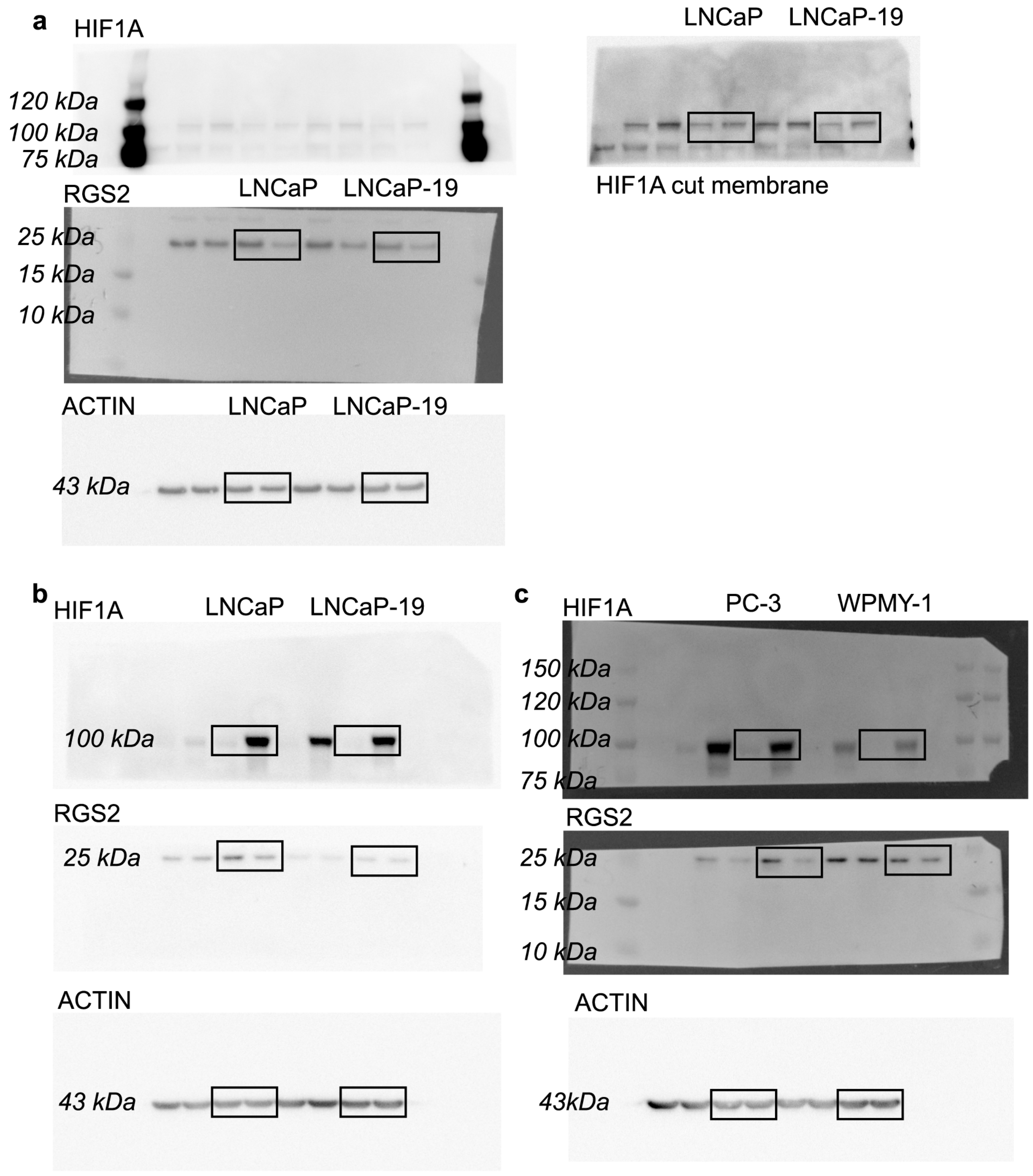

Figure S3 ***Original blots from Fig 2.***
a Blots of experiments carried out at 1% oxygen level with corresponding control for 48 and 72 hours. **b** $CoCl_2$ treatment (150 µM) of LNCaP and LNCaP-19 for 48 and 72 hours. **c** Corresponding treatment of PC-3 and WPMY-1 cells. Squares marks the samples included in the article (72 hours exposure).

Supplementary Figure S4 *RGS2 knockdown effects on signalling pathways associated with PC progression*

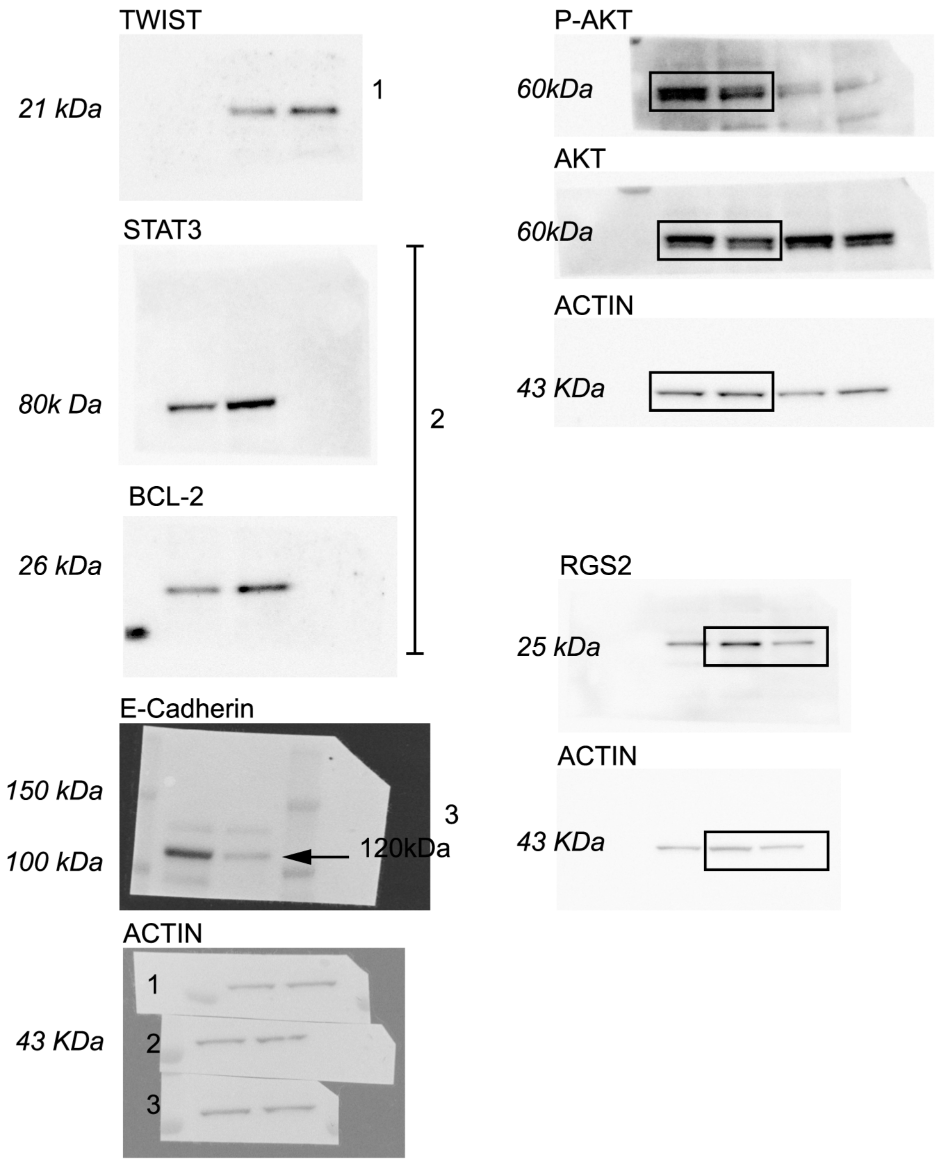

Figure S4 ***Original blots from Fig 5 and 6.***
Squares marks the samples included in the article. Molecular markers were included for clarity. Blots are grouped according to gel affiliation. Loading control ACTIN was matched by number to the corresponding blot(s).

Supplementary Figure S5 *Evaluation of RGS2 expression in PC cell lines.*

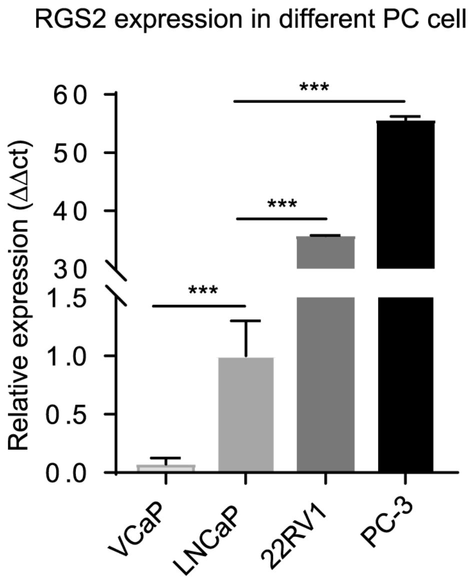

Figure S5 ***Relative gene expression of RGS2 in comparison to LNCaP.***
